# Competitive dynamics underlie cognitive improvements during sleep

**DOI:** 10.1073/pnas.2109339118

**Published:** 2021-12-13

**Authors:** Pin-Chun Chen, Hamid Niknazar, William A. Alaynick, Lauren N. Whitehurst, Sara C. Mednick

**Affiliations:** ^a^Department of Cognitive Sciences, University of California, Irvine, CA 92697;; ^b^Department of Statistics, University of California, Irvine, CA 92697;; ^c^ScholarNexus LLC, San Diego, CA 92122;; ^d^Department of Psychology, University of Kentucky, Lexington, KY 40506

**Keywords:** working memory, long-term memory, sleep, vagal activity, spindle activity

## Abstract

We provide evidence that human sleep is a competitive arena in which cognitive domains vie for limited resources. Using pharmacology and effective connectivity analysis, we demonstrate that long-term memory and working memory are served by distinct offline neural mechanisms that are mutually antagonistic. Specifically, we administered zolpidem to increase central sigma activity and demonstrated targeted suppression of autonomic vagal activity. With effective connectivity, we determined the central activity has greater causal influence over autonomic activity, and the magnitude of this influence during sleep produced a behavioral trade-off between offline long-term and working memory processing. These findings suggest a sleep switch mechanism that toggles between central sigma-dependent long-term memory and autonomic vagal-dependent working memory processing.

## Significant

Sleep facilitates both long-term episodic memory consolidation and short-term working memory functioning. However, the mechanism by which the sleeping brain performs both complex feats and which sleep features are associated with these processes remain unclear. Using a pharmacological approach, we demonstrate that long-term and working memory are served by distinct offline neural mechanisms and that these mechanisms are mutually antagonistic. We propose a sleep switch model in which the brain toggles between the two memory processes via a complex interaction at the synaptic, systems, and mechanistic level with implications for research on cognitive disturbances observed in neurodegenerative disorders such as Alzheimer’s and Parkinson's disease, both of which involve the decline of sleep.

Working memory (WM) and long-term memory (LTM) serve separate functions, and the idea that they are supported by separate systems has become a core assumption of modern cognitive psychology (1). WM is a control process for planning and carrying out behavior that is information-independent, whereas LTM is an information-dependent vast store of knowledge and record of prior events. Both WM and LTM rely on offline periods that include sleep to facilitate performance improvement. According to the framework of systems consolidation, LTMs are initially bound by a fast-learning system in the hippocampus (i.e., encoding) and followed by a stabilization of these memory traces in cortical stores (i.e., consolidation). Non-rapid eye-movement (NREM) sleep may facilitate consolidation by increasing communication between cortico-thalamo-hippocampal circuits via nested oscillations of slow oscillations (<1 Hz, SO), spindles (sigma power; 12 to 15 Hz), and sharp wave ripples (SW-Rs), respectively (2–4). SOs reflect fluctuations of the membrane potential and orchestrate transitions from neuronal silence (hyperpolarized down states) to neuronal excitation (depolarized up states). Spindles, nested in SO up states, gate dendritic Ca^2+^ influx and promote synaptic plasticity. Hippocampal SW-Rs nested in spindles are closely linked to the reactivation of cell assemblies engaged during encoding. Prior studies suggested that spindles may initiate hippocampal–cortical dialogue by grouping SW-Rs, which facilitates information transfer between neocortical and hippocampal cell assemblies. In humans, pharmacological interventions that boost spindle activity enhance sleep-dependent hippocampal LTM, measured by the paired-associates task (5–7).

Classic models of WM propose two governing mechanisms: 1) an active maintenance of information online through the elevated firing of prefrontal neurons and 2) a supervisory executive control process that is supported by a prefrontal–subcortical inhibitory network (8, 9). Due to innervations to the heart via sympathetic stellate ganglia and parasympathetic vagal nerve efferents, cardiac autonomic activity is thought to reflect functioning of prefrontal inhibitory processing (10). Accordingly, vagally-mediated, high-frequency heart rate variability (HF HRV, 0.15 to 0.40 Hz) during wake correlates with executive function tasks, such as WM, which rely on the prefrontal cortex (PFC) (11). Improvement in WM, however, only occurs when the interval between training sessions contains a period of sleep, measured by *N*-back, complex-span task, and digit span (12–17). Although the exact mechanisms of WM improvement during sleep are still not entirely understood, prior studies point to slow-wave sleep (SWS) as an optimal state for synaptic plasticity and cortical reorganization. During SWS, vagal activity is also at its highest compared to all other states of consciousness (18). Building on this foundation, a recent study identified vagal HF HRV during SWS as a strong predictor of WM improvement, measured by the operation-span (OS) task (19).

Together, theoretical models and empirical data suggest that NREM sleep may facilitate improvement in WM via strengthening of prefrontal–autonomic inhibitory networks, measured by HF HRV, while facilitating the formation of LTM via thalamic spindles driving the hippocampal–cortical dialogue, measured by sigma power. The question is how the sleeping brain performs both of these complex feats and which sleep features are associated with these processes. Prior animal studies suggest a potentially antagonistic interplay between the cortico-thalamo-hippocampal networks and the prefrontal–autonomic inhibitory networks (20, 21). However, this possibility and its functional significance have not been studied in humans.

In the present study, we enacted a pharmacological strategy to investigate the bidirectional interplay between central (reflected in sigma activity) and autonomic (reflected in vagal HRV) activities during overnight sleep and its impact on LTM and WM, measured by the word-paired associative (WPA) task and the OS task. Specifically, we tested our model that central sigma activity would suppress autonomic vagal activity using effective connectivity (22), defined as the influence that one neural system exerts over another, which can be estimated using Granger causality (see Fig. 3*A*). We identified an antagonistic relationship between sigma and vagal activity during sleep with the degree of mutual antagonism between sigma and vagal activity predicting a behavioral trade-off between LTM and WM. These results suggest that NREM sleep confers benefits to WM and LTM by switching between separate offline mechanisms (i.e., the prefrontal–autonomic inhibitory processing and the hippocampal–cortical dialogue). Furthermore, this sleep switch can be biased toward LTM consolidation by increasing sigma activity, in this case pharmacologically, and presumably by other methods as well. These results illuminate the dynamics interplay underlying LTM and WM processes during sleep.

## Results

### Experiment 1.

**Fig. 1.**
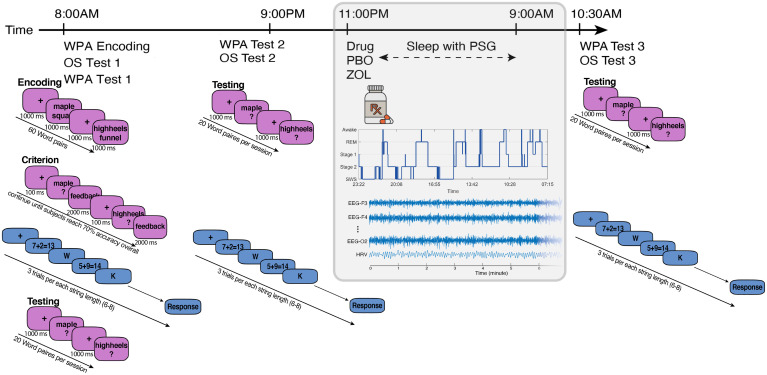
Experimental design and behavioral tasks. Experiment 1: Participants reported to the laboratory at 9:00 PM and were hooked up to polysomnography, including EEG, ECG, EMG, and EOG. Before sleep, we recorded 5-min resting HRV while subjects lay awake in a still, supine position. At 11:00 PM, directly before lights out, subjects ingested either 10 mg zolpidem or placebo. Sleep was monitored online by a trained sleep technician. Participants were woken up at 9:00 AM the next morning and permitted to leave the laboratory. Each participant experienced two visits per drug condition (a total of four visits). Experiment 2: At 8:00 AM, participants began encoding for the episodic memory WPA task followed by the WM OS task and immediate recall for the WPA (Test 1). Participants left the laboratory after cognitive testing. Participants were asked not to nap, exercise, or consume caffeine or alcohol and were monitored with actigraphy during the break. Participants returned to the laboratory at 9:00 PM to complete the delayed recall over wake for WPA and OS (Test 2). Participants were then hooked up to polysomnography, including EEG, ECG, EMG, and EOG. Before sleep, we recorded 5-min resting HRV while subjects lay awake in a still, supine position. At 11:00 PM, directly before lights out, subjects ingested either 10 mg zolpidem or placebo. Sleep was monitored online by a trained sleep technician. Participants were woken up at 9:00 AM the next morning and provided a standardized breakfast. At 10:30 AM, participants completed the delayed recall over sleep for WPA and OS (Test 3). For both tasks, to assess the change in performance, we measured two difference scores: overnight change (Test 3 to Test 2) and 24-h change (Test 3 to Test 1). Each participant experienced one visit per drug condition (a total of two visits). See *SI Appendix*, Fig. S1 and Table S12 for summary statistics.

Based on previous findings, we predicted that central sigma power would have an inhibitory effect on cardiac vagal tone. To this end, we administered zolpidem in a double-blind, placebo-controlled, randomized crossover design in which each participant experienced two nights per drug condition (zolpidem or placebo; a total of four nights; *n* = 34; *M*_age_ = 20.88 ± 1.88 y, 17 females), with electroencephalography (EEG) and electrocardiogram (ECG) monitoring (Fig. 1, shaded area). The order of drug conditions was counterbalanced with at least a 1-wk interval between the experimental visits to allow for drug clearance. We performed power spectral analysis to quantify normalized sigma activity and analyzed HRV profiles. Our intervention was successful, whereby zolpidem increased time spent in SWS while decreasing wake after sleep onset (WASO) (*SI Appendix*, Table S2) and enhanced sigma activity during Stage 2 sleep (central channels: t = 2.112, *P* = 0.0349; parietal channels: t = 2.214, *P* = 0.0270, corrected by Tukey’s multiple comparisons; *SI Appendix*, Table S7), consistent with prior literature.

**Fig. 2.**
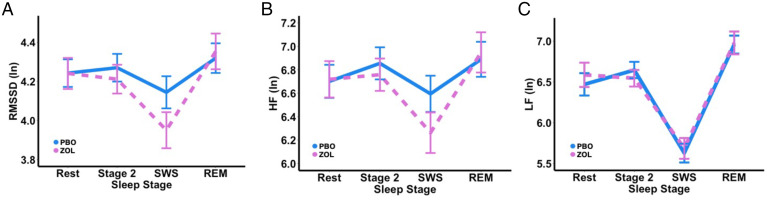
Zolpidem decreased vagally mediated HRV, but not LF, during SWS. (*A*) RMSSD: We report a significant main effect of sleep stage [*F*(3, 366) = 21.257, *P* < 0.0001] with a decreased HRV during SWS compared to Rest, Stage 2, and REM (all *P*s < 0.0001). We also found a significant interaction [*F*(3, 366) = 3.8630, *P* = 0.0096] between sleep stage and drug condition with decreased vagal activity during SWS (*P* = 0.0006) in zolpidem compared with placebo but not during Stage 2 (*P* = 0.3549), REM (*P* = 0.3804), or Rest (*P* = 0.6152). The LRT was significant (LR = 13.8544; *P* = 0.0078), suggesting that zolpidem significantly modulated the time-domain measure of HRV. (*B*) HF HRV: We report a significant main effect of sleep stage [*F*(3, 366) = 16.9891, *P* < 0.0001] with a decreased HRV during SWS compared to Rest (*P* = 0.0006), Stage 2 (*P* < 0.0001), and REM (*P* < 0.0001). Similarly, we also report a significant interaction [*F*(3, 366) = 3.1899, *P* = 0.0238] between sleep stage and drug condition with decreased vagal activity during SWS (*P* = 0.0020) in zolpidem compared to placebo but not during Stage 2 (*P* = 0.4194), REM (*P* = 0.4365), or Rest (*P* = 0.6070). The LRT was significant (LR = 11.3671; *P* = 0.0227), suggesting that zolpidem significantly modulated the frequency-domain measure of HRV. (*C*) LF HRV: We report a significant main effect of sleep stage [*F*(3, 366) = 93.0330, *P* < 0.0001] with a decreased LF power during SWS compared to Rest, Stage 2, and REM (all *P*s < 0.0001) and an increased LF power during REM compared to Rest and Stage 2 (all *P*s < 0.0001). No significant main effect of drug condition (*P* = 0.6337) nor interaction between sleep stage and drug condition (*P* = 0.5681) were found. The LRT was not significant (LR = 2.2889; *P* = 0.6828), suggesting that zolpidem did not significantly modulate low frequency HRV.

As we hypothesized, zolpidem not only increased sigma activity but also selectively suppressed the vagal tone during sleep measured by rms of the successive differences (RMSSD) (Fig. 2*A*) and HF HRV (Fig. 2*B*) but had no impact on low-frequency (LF) HRV (0.04 to 0.1; Fig. 2*C*). Other HRV indices were reported in *SI Appendix*, Fig. S2 and Table S4.

**Fig. 3.**
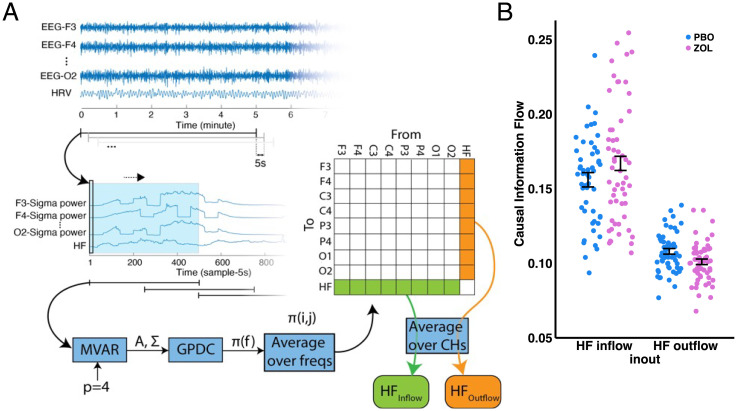
Effective connectivity modulated by drug condition. (*A*) Effective Connectivity Estimation Procedure (see Materials and Methods for details): Prior research using functional connectivity analysis has measured temporal similarity or correlations between different EEG channels (57). Although functional connectivity can reveal important information about communication, it is limited to correlational measures and cannot identify directional causal communication. In contrast, effective connectivity is defined as the influence that one neural system exerts over another either directly or indirectly (22), which can be estimated using the Granger causality (58). According to the Granger causality, a causal relation is detected if past values of a source signal help predict a second signal (sink signal) beyond the information contained in its past alone. Granger causality and causal information flow can be quantified using an MVAR and then examining the coefficients of the fitted model. Partial directed coherence quantifies direct causal information outflow from each signal to all other signals, emphasizing the sinks rather than the sources (59). The current study adopted GPDC to quantify causal information flow (60) with respect to both the source and the sink regions. The model order (p) of the MVAR model was the only parameter and was selected based on the Akaike criterion. (*B*) Experiment 1 Effective Connectivity: We report a main effect of inflow versus outflow [*F*(1, 185) = 273.317, *P* < 0.0001) with a greater HFinflow than HFoutflow in both drug conditions; an interaction between drug condition and inflow versus outflow [*F*(1, 185) = 5.744, *P* = 0.0175] with a greater HFinlfow during zolpidem compared to placebo (*P* = 0.0369). No main effect of drug condition was found [*F*(1, 185) = 0.512, *P* = 0.4751]. The LRT was significant (LR = 6.0745; *P* = 0.0480), suggesting that zolpidem significantly modulated the causal information flow between sigma and HF activity. Effective connectivity ratios (HFInflow*/*HFOutflow) increased significantly during the zolpidem night [*F*(1, 79) = 8.0607, *P* = 0.0059].

We then tested our hypothesis that central sigma power would exert greater causal influence over vagal autonomic activity than the influence of vagal over sigma activity, and that such a difference would be increased by zolpidem. To test this prediction, we used the effective connectivity estimation (Fig. 3*A*). In particular, we tested the hypotheses that central sigma naturally exercises greater causal influence on autonomic vagal activity than vice versa in the placebo condition, and that increasing sigma with zolpidem would increase causal information flow from sigma to vagal activity while decreasing the causal information flow from vagal to sigma activity in the zolpidem condition. For each subject, we calculated two measures: HFInflow and HFOutflow, respectively (see *Materials and Methods*). We confirmed our hypothesis that central sigma power exerted greater flow on vagal activity than vice versa in the placebo condition (HFInflow > HFOutflow; *P* < 0.0001; Fig. 3*B*). We also confirmed that such difference was increased by zolpidem (*P* = 0.0369; Fig. 3*B*). Next, we calculated a composite score, the effective connectivity ratio: HFInflow over HFOutflow, in which higher numbers represented greater central sigma control over autonomic vagal activity. We observed a higher effective connectivity ratio during the zolpidem night (*P* = 0.0059). Taken together, results from Experiment 1 were consistent with our hypotheses that central sigma activity naturally exerts dominance over autonomic activity during NREM sleep, and that increasing sigma activity via zolpidem inhibits vagal activity and enhances central sigma control over autonomic vagal activity.

### Experiment 2.

In an independent sample of participants (*n* = 38; *M*_age_ = 20.85 ± 2.97 y; 19 females), we added a behavioral experiment (Experiment 2; Fig. 1) to the original design of Experiment 1 to test if we could replicate the physiological results of Experiment 1 and determine their functional importance for sleep-dependent cognition. Again, we exploited zolpidem to modulate the interaction between central sigma and autonomic vagal activity and examined its impacts on the improvements of LTM and WM (Fig. 1). The order of drug conditions was counterbalanced with at least a 1-wk interval between the two experimental visits to allow for drug clearance. The goal of Experiment 1 was to thoroughly describe the physiological phenomenon across the whole night, whereas the goal for Experiment 2 was to examine the functional impact of the pharmacological intervention on performance. For this reason, in Experiment 2, we divided the night into quartiles and focused our analyses on quartile two and three combined to maximize zolpidem’s effect because of the pharmacodynamics of zolpidem, which has a half-life of 1.5 to 4.5 h, and onset [mean Tmax 1.6 h (23)]. We hypothesized that sigma-guided vagal suppression effects would result in parallel behavioral effects, with greater LTM and reduce improvement in WM. We further hypothesized that the magnitude and the direction of causal information flow between central and autonomic systems would be correlated with the trade-off between LTM and WM.

The physiological results across one night of sleep in Experiment 2 were consistent with those from two nights of sleep in Experiment 1 (see *SI Appendix*, Table S3 for sleep architecture, Table S8 for power spectrum, Figs. S3–S6 and Tables S5 and S6 for HRV, and Fig. S7 for effective connectivity). We confirmed that zolpidem increased sigma activity during sleep while suppressing vagal tone (measured by RMSSD and HF HRV), but had no impact on LF HRV. Similarly, we replicated the effective connectivity results (Fig. 3*C*) in which zolpidem increased the effective connectivity ratio (*P* = 0.0265), indicating greater causal influence of central sigma activity on autonomic vagal activity.

**Fig. 4.**
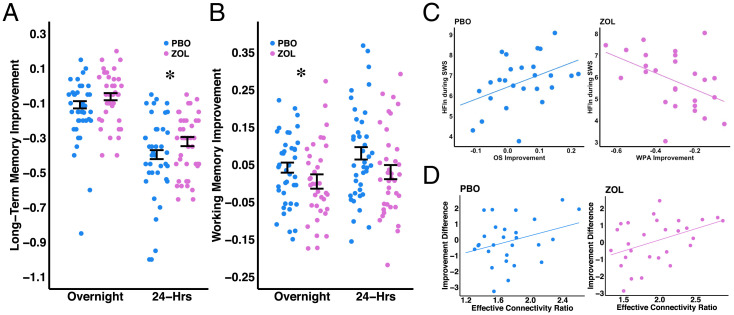
Zolpidem increases LTM but decreases WM improvement. (*A*) LTM (WPA task) improvement by drug conditions and time (*y*-axis: WPA overnight [Test 3 to Test 2] and 24-h [Test 3 to Test 1] improvement; asterisks indicate significant differences in behavioral changes between two drug conditions; *P < 0.05). Zolpidem (ZOL) yielded greater but not significant overnight retention of WPA than the placebo (PBO) condition (estimate= −0.1156, CI= (−0.2408, −0.0095), t = −1.8104, *P* = 0.0810), accounting for visit, as well as greater 24-h retention of WPA than PBO visits (estimate = −0.1810, CI = [−0.3519, −0.0096], t = −2.0704, *P* = 0.0474) accounting for visit. (*B*) WM (OS task) improvement by drug conditions and time (*y*-axis: OS overnight [Test 3 to Test 2] and 24-h [Test 3 to Test 1] improvement; asterisks indicate significant differences in behavioral changes between two drug conditions; *P < 0.05). PBO showed significantly greater overnight improvement of OS than ZOL visits (estimate = 0.1242, CI = [0.0201, 0.2284], t = 2.3377, *P* = 0.0260), accounting for Test 2 performance and visit as well as greater but not significant 24-h improvement of OS than ZOL visits (estimate = 0.1000, CI = [−0.0184, 0.2185], t = 1.6546, *P* = 0.1081), accounting for Test 1 performance and visit. (*C*) Functional role of vagal activity on memory (*y*-axis: HFln during SWS, *x*-axis: OS overnight and WPA 24-h improvement). Vagal activity during SWS positively predicted working memory (OS task) improvement (*r* = 0.422; *P* = 0.032) but negatively predicted long-term memory (WPA task) improvement (r = −0.460; *P* = 0.018). The difference between these two correlations was significant (Z = 3.67; *P* = 0.0001). (*D*) Functional role of effective connectivity ratio on memory trade-off (*y*-axis: normalized WPA improvement to normalized OS improvement score, *x*-axis: effective connectivity ratio = HFInflow*/*HFOutflow). The effective connectivity ratio (a higher ratio indicates a greater causal effect from sigma to vagal) during sleep positively predicted memory trade-off (a greater difference indicates a greater improvement in the WPA task than the OS task) during the zolpidem night (*r* = 0.429; *P* = 0.020) but not the placebo night (*r* = 0.251; *P* = 0.190). The difference between these two correlations is not significant (Z = 0.78; *P* = 0.2177).

We further assessed the functional roles of each physiological measure (EEG sigma activity, cardiac vagal activity, and the effective connectivity ratio) on LTM and WM changes across sleep. We hypothesized that increasing sigma activity would benefit LTM retention in a WPA task, whereas decreasing vagal activity would hinder WM improvement on a WM OS task. To this end, we examined overnight and 24-h change scores in each task between the two drug conditions. For the WPA task, our analysis showed that zolpidem significantly increased 24-h LTM retention (Fig. 4 *A*, *Right*) and overnight retention (Fig. 4 *A*, *Left*). For the WM OS task, our analysis demonstrated that zolpidem decreased overnight improvement (Fig. 4 *B*, *Left*) and 24-h improvement (Fig. 4 *B*, *Right*) compared to placebo. In summary, we confirmed our behavioral hypothesis that sigma-guided vagal suppression would increase LTM (Fig. 4*A*) and decrease WM improvement (Fig. 4*B*).

Next, we tested the correlations between each physiological measure (EEG sigma activity, cardiac vagal activity, and the effective connectivity ratio) and memory changes across sleep using Pearson’s correlation coefficients. We found a functional dissociation in vagal activity and behavior in which vagal activity during SWS was negatively correlated with LTM in the zolpidem condition (24-h retention and HF HRV: r = −0.460; *P* = 0.018; Fig. 4 *C*, *Right*) and positively correlated with WM improvement (overnight retention and HF HRV: *r* = 0.422; *P* = 0.032; Fig. 4 *C*, *Left*) in the placebo condition. We compared correlations between HFln and LTM versus HFln and WM, and the difference was significant (Z = 3.67; *P* = 0.0001). This result is in line with our expectation that vagal activity during sleep differentially supports LTM and WM. Correlational statistics between vagally mediated HRV parameters and behavioral improvements are shown in *SI Appendix*, Table S9. No significant correlations were found between EEG sigma activity and WM improvement (zolpidem: all *P*s > 0.5687; placebo: all *P*s > 0.1943) or between EEG sigma activity and LTM retention (zolpidem: all *P*s > 0.15516; placebo: all *P*s > 0.1383; see *SI Appendix*, Fig. S8 for spindle density). Taken together, vagal activity was positively associated with WM improvement but inversely related to LTM.

We then asked if central and autonomic antagonism impacted the trade-off between LTM and WM improvement by correlating the effective connectivity ratio with the normalized LTM–WM difference score in which higher numbers represent greater LTM than WM improvement. We found a positive correlation between the effective connectivity ratio and the normalized LTM–WM difference score in the zolpidem (*r* = 0.429; *P* = 0.020; Fig. 4 *D*, *Right*) and nonsignificant positive correlation in the placebo condition (*r* = 0.251; *P* = 0.190; Fig. 4 *D*, *Left*). These results suggested that the more central activity exerted influence on autonomic vagal activity, the more sleep was biased toward sigma-dependent LTM consolidation (and away from vagal-dependent WM processing). We further compared correlations between the LTM–WM difference score and the effective connectivity ratio in the placebo versus zolpidem condition. The difference was not significant (Z = 0.78; *P* = 0.2177), suggesting that zolpidem amplified the natural vagal suppression by sigma and thus increased the magnitude of the correlations.

**Fig. 5.**
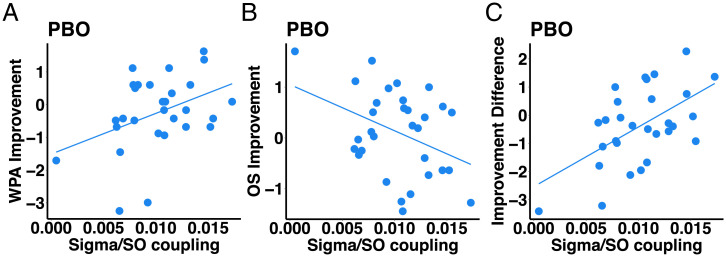
Functional roles of sigma power coupled with SO up state on LTM and WM. (*A*) LTM (WPA task) improvement positively correlated with sigma power coupled with an SO up state (*y*-axis: normalized score of WPA 24-h improvement; *x*-axis: normalized sigma power coupled during the up state of SOs; *r* = 0.400; *P* = 0.034). (*B*) WM (OS task) improvement negatively correlated with sigma power coupled with SO up state (*y*-axis: normalized score of OS overnight improvement; *x*-axis: normalized sigma power coupled during the up state of SOs; r = −0.380; *P* = 0.033). (*C*) Improvement difference positively correlated with sigma power coupled with SO up state (*y*-axis: normalized WPA improvement to normalized OS improvement score; *x*-axis: normalized sigma power coupled during the up state of SOs; *r* = 0.560; *P* = 0.002).

Given the critical role for system consolidation of nested oscillations between sigma and SOs, the current findings led us to the prediction that greater sigma–SO coupling would evince increased LTM via suppressed WM. We tested this prediction by computing sigma power during the up state of SOs and correlating this magnitude with the normalized LTM–WM improvement difference score (see *SI Appendix*, Table S10 for SO counts and sigma/SO summary statistics and Table S11 for correlations). We found that zolpidem decreased the number of SOs, a finding consistent with prior literature that zolpidem shifts brain activity to faster frequencies. This decrease in SOs by zolpidem led us to examine coupling in the placebo condition in which we found a significant positive correlation between sigma power during SOs up state and difference in LTM–WM improvement (Fig. 5), consistent with the notion that competitive dynamics underlie the fundamental mechanisms of cognitive improvements during sleep.

## Discussion

The current work identified two neural mechanisms during NREM sleep that support the distinct enhancements in LTM and WM. In Experiment 1, we exploited the hypnotic zolpidem to enhance sigma activity during NREM sleep and reported that increasing sigma activity resulted in targeted vagal suppression during NREM. Next, we used the effective connectivity estimation technique to test the causal hypothesis that central sigma activity actively suppressed vagal autonomic activity. Consistent with our hypothesis, the results showed that central sigma exerted greater causal control over autonomic vagal activity and that pharmacologically increasing sigma activity boosted causal information flow from central to autonomic channels and decreased flow from autonomic to central channels. In a separate set of subjects, we replicated the pharmacological intervention and tested the functional significance of the sigma–vagal mutual antagonism during NREM sleep by testing LTM and WM before and after a night of sleep. The physiological and effective connectivity results replicated those of Experiment 1. Moreover, the sigma-guided vagal suppression was associated with enhanced LTM retention at the cost of reduced WM improvement. Additionally, the magnitude of vagal suppression as well as the degree of sigma–SO coupling predicted a not previously reported trade-off between LTM and WM processing. These findings suggest evidence for a sleep switch that toggles between separate and nonoverlapping NREM mechanisms that support LTM and WM processing. Furthermore, this switch can be biased toward greater LTM consolidation by boosting sigma activity.

Sigma activity is proposed to facilitate plasticity by producing long-term changes in responsiveness in cortical neurons (24) and increasing dendritic Ca^2+^ influxes (25), particularly enhanced when coupled to down-to-up transitions of the sleep SO. Recently, Dickey and colleagues demonstrated that sigma activity may promote spike timing–dependent plasticity, which facilitates long-term potentiation, the cellular mechanism thought to underlie learning and memory (26). Thus, sigma activity may promote LTM via cellular synaptic plasticity. Furthermore, at the systems level, sigma nested within SOs may also support the replay of memory traces during consolidation (27), and causally increasing sigma activity boosts hippocampal-dependent memory consolidation (5, 28, 29). The current findings demonstrate that sigma activity, especially when coupled with SOs, also suppresses subcortical vagal activity with significant functional outcomes, specifically a reduction in WM.

Vagal influence on cognitive function is a core principle of the Neurovisceral Integration Model (10), which posits that autonomic activity is a peripheral index of the integrity of prefrontal–autonomic networks that support inhibitory, goal-directed, high-order brain functions. The 10th cranial vagus nerve communicates peripheral information to and from the brainstem, with afferents projecting to higher-order, cognitive areas such as the prefrontal cortex, anterior cingulate, and amygdala. Additionally, descending projections from the PFC to the brainstem and hypothalamic structures allow for bidirectional communication between the central nervous system and the autonomic nervous system through the vagus nerve (10). As such, high levels of vagally mediated HRV are associated with superior executive function (30), WM (11), and emotional regulation (31). Cognitive training including WM has demonstrated that vagal activity reflects enhanced cognitive control of prefrontal networks (32). Although sleep is not typically measured across the cognitive training interventions, the current findings suggest that executive function improvement may be mediated by the strengthening of prefrontal–autonomic networks during sleep.

Parasympathetic vagal activity is highest during SWS compared to all other states of consciousness (33). Vagal activity is strongly coupled with delta activity (<4 Hz) during SWS, and vagal enhancement precedes the onset of SWS (34). Several studies have linked SOs with WM improvement. For example, studies have shown that frontoparietal SOs, but not sigma, predict WM improvement (16, 35). However, not all studies report a consistent association between SOs and WM (13, 36, 37), and few account for autonomic activity. Chen and colleagues reported that vagal activity during SWS was a better predictor of WM improvement than SOs or vagal activity during wake (19). In the current work, we found that changes in vagal autonomic activity during SWS, but not SOs per se, were critical for WM performance improvement. This, together with prior findings, suggests a nonnegligible role of vagal influence on WM plasticity.

Given that both LTM and WM appear to rely on NREM sleep, one clear question emerges: How are the limited resources of NREM sleep shared across cognitive processes? The current findings are consistent with the hypothesis that competitive neural dynamics during NREM sleep underlie cognitive improvement. Supporting this hypothesis, prior research has shown that vagal nerve stimulation activates neurons in the locus coeruleus (LC) and increases norepinephrine (NE) levels in the brain (38, 39), and inactivation of LC impairs WM acquisition while having no effect on consolidation or retention of spatial memories (40–44), whereas up-regulating GABAergic networks impaired WM performance (45). On the other hand, using ripple-triggered fMRI in monkeys, Logothetis and colleagues demonstrated that ripples orchestrate a privileged state of enhanced central brain activity by silencing output from the diencephalon, midbrain, and brainstem, regions associated with autonomic regulation, which may serve to boost communication between the hippocampus and cortex (20). In addition, in both humans and mice, Lecci et al. demonstrated that heart rate and sigma power oscillate in antiphase with each other at 0.02 Hz, suggesting a periodic switch between sigma and autonomic activation every 50 s (46).

Here, using effective connectivity, we demonstrated that a GABAergic agonist enhanced naturally occurring cortical sigma dominance over vagal autonomic activity. Similar vagolytic findings have been shown with zolpidem in persistent vegetative state patients (47). Furthermore, the magnitude of this central sigma influence on vagal activity predicted the trade-off between overnight LTM and WM improvement. Together with the previous literature, these finding suggest that sigma-dependent processes, including GABAergic hippocampal–thalamocortical networks, and vagal-dependent processes, including noradrenergic frontal–autonomic networks, may compete for sleep resources during NREM sleep. We hypothesize that the shared resource may be the SOs, which, when coupled with ripple-nested sigma, promotes LTM and suppresses other processes and, when uncoupled, facilitates WM by enhancing prefrontal–autonomic networks. We further hypothesize that sigma may act as a gating mechanism that regulates SO resources for other processes, which would explain the mixed findings of SOs for WM improvement. Given that ∼20% of SOs during NREM are sigma coupled (48), this leaves plenty of resources to be divided among other processes, including WM.

**Fig. 6.**
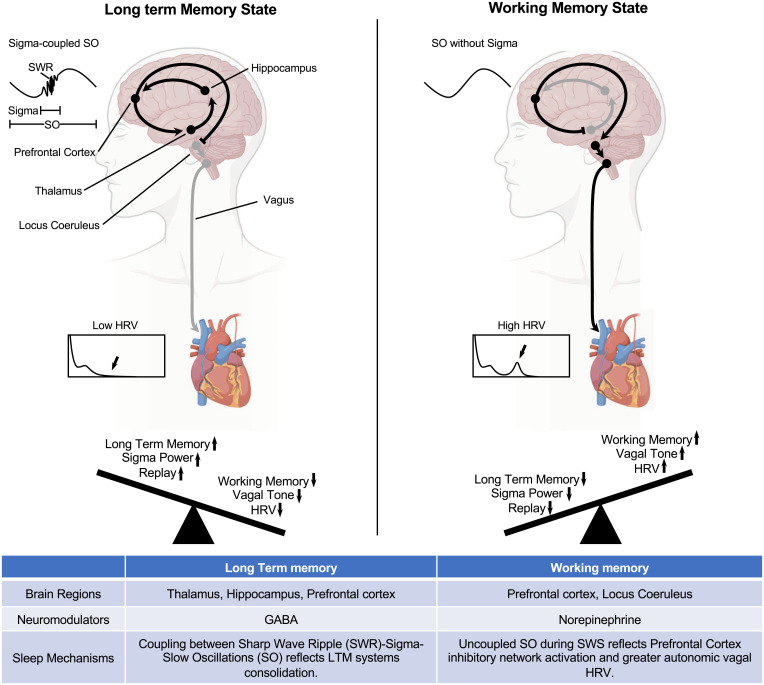
Sleep switch model. The model represents the proposed brain regions, primary neuromodulators, and sleep mechanisms involved in the LTM state and the WM state that toggle throughout NREM sleep. During the LTM state, consolidation occurs via sigma-coupled SOs, which leads to reduced autonomic vagal-dependent activity and less WM improvement. During the WM state, greater efficiency occurs during uncoupled SOs associated with increased autonomic vagal-dependent activity, which leads to reduced central sigma-dependent activity and less LTM consolidation.

These data suggest a trade-off in which the two memory processes (LTM and WM) alternate during NREM sleep via a complex interaction at the synaptic (GABA versus NE activation), systems (thalamocortical versus frontal midbrain), and mechanistic level (sigma-coupled SO versus uncoupled SO) (see the graphical model in Fig. 6). Further research enhancing vagal activity and suppressing sigma activity is needed to show a double dissociation and tease apart these competitive mechanisms. Future work is also required to test the generalizability across multiple cognitive domains (i.e., motor learning) and tasks (i.e., nonassociative LTM and *N*-back WM tasks) that rely on NREM sleep. The sleep switch mechanism and separable sleep features associated with WM and LTM processing suggest directions for future translational research on cognitive disturbances observed in neurodegenerative disorders such as Alzheimer’s and Parkinson's disease, both of which involve the decline of sleep (49, 50).

### Limitations and Future Research.

Limitations of this study include using a convenience sample of both men and women and a lack of hormonal status among the young women, which can have an impact on cardiac vagal activity (51) and sigma activity (52). Future studies examining hormonal fluctuation are needed to understand the interaction between central sigma and autonomic profiles during sleep and their impact on cognition. Additionally, though we did not measure respiration directly, we did analyze the frequency peak of HF (HFfp) in order to control for respiratory rate, which can affect the HRV. HFfp showed no difference between the two drug conditions and varied within a narrow range in the HF spectrum, between 0.22 and 0.26 Hz. Thus, it is unlikely that respiratory activity played a key role in zolpidem’s modulation on HRV and memory. However, we cannot completely exclude the effect of drugs on cardiopulmonary coupling as may be detected using measures of coherence. In addition, our experimental design did not include an adaptation night and thus may have caused the “first-night effect.” However, the visits were counterbalanced by drug conditions; therefore, the first-night effect should have canceled out across subjects. Furthermore, given that zolpidem is commonly prescribed to insomniacs, studies are needed to investigate if chronic use of zolpidem leads to WM deficits or biased memory trade-off during sleep. Lastly, because of methodological differences between EEG and ECG analyses, we measured sigma power as a proxy of spindles, which was not directly correlated with sleep-dependent behavioral changes. In addition, our study was limited by adhering to standard measures of vagal activity that require 5-min epochs, which reduced temporal specificity. This limitation constrains our effective connectivity analysis to all sleep epochs. We reported rapid eye movement (REM) and wake-free results in *SI Appendix* (*SI Appendix*, Figs. S9 and S10), which were in the same direction as what we reported in the main paper but less robust, likely because of under power (*SI Appendix*, Table S13). It is therefore crucial that future research develops validated markers of vagal activity in shorter windows. Our results are a lack of temporal specificity of sleep micro events, and thus, future research with a greater temporal precision around physiological events is needed to provide insight into shifts between central- and autonomic-dependent activities.

## Materials and Methods

### Participants.

A total of 34 adults in Experiment 1 (*M*_age_ = 20.88 ± 1.88 y, 17 females) and 38 adults in Experiment 2 (*M*_age_ = 20.85 ± 2.97 y, 19 females) with no history of neurological, psychological, or other chronic illnesses were recruited for the study (*SI Appendix*, Table S1, Demographics). All participants signed informed consent, which was approved by the Western Institutional Review Board and the University of California, Riverside, Human Research Review Board. Exclusion criteria included irregular sleep/wake cycles, sleep disorder, personal or familial history of diagnosed psychopathology, substance abuse/dependence, loss of consciousness greater than 2 min or a history of epilepsy, current use of psychotropic medications, and any cardiac or respiratory illness that may affect cerebral metabolism, which was determined during an in-person psychiatric assessment with trained research personnel. Additionally, all participants underwent a medical history and physical appointment with a staff physician to ensure their physical wellbeing. All subjects were naive to or had limited contact with (less than two lifetime use and no use in last year) the medication used in the study. Participants were asked to refrain from consuming caffeine, alcohol, and all stimulants for 24 h prior to and including the study day. Participants filled out sleep diaries for 1 wk prior to each experiment and wore wrist-based activity monitors the night before the study (Actiwatch Spectrum, Philips Respironics) to ensure participants were well rested (at least 7 h per night during the week including the eve of the experiment day). Participants received monetary compensation and/or course credit for participating in the study. Study procedures were illustrated in Fig. 1.

### Data Reduction.

#### Experiment 1.

A total of 25 participants completed four visits (two placebo nights and two zolpidem nights), eight participants completed two visits (one placebo night and one zolpidem night), and one participant completed a zolpidem visit because of scheduling conflicts. Therefore, 56 placebo and 59 zolpidem nights were included in the analyses.

#### Experiment 2.

A total of 36 participants completed the placebo night, and 35 participants completed the zolpidem night polysomnography recordings. A total of 35 participants completed all three sessions of OS (WM) tasks in both placebo and zolpidem conditions. A total of 33 participants completed all three sessions of the WPA (LTM) task in both placebo and zolpidem conditions.

### Sleep Recording.

EEG data were acquired using a 32-channel cap (EASEYCAP GmbH) with Ag/AgCI electrodes placed according to the international 10 to 20 System (Jasper, 1958). A total of 22 electrodes were scalp recordings, and the remaining electrodes were used for ECG, electromyogram (EMG), electrooculogram (EOG), ground, an online common reference channel (at FCz location, retained after rereferencing), and mastoid (A1 and A2) recordings. The EEG was recorded with a 1,000-Hz sampling rate and was rereferenced to the contralateral mastoid (A1 and A2) postrecording. The data were preprocessed using BrainVision Analyzer 2.0 (BrainProducts). Eight scalp electrodes (F3, F4, C3, C4, P3, P4, O1, and O2), the EMG, and the EOG were used in the scoring of the nighttime sleep data. High-pass filters were set at 0.3 Hz and low-pass filters at 35 Hz for EEG and EOG. Raw data were visually scored in 30-s epochs into Wake, Stage 1, Stage 2, SWS, and REM sleep according to the Rechtschaffen and Kales’ manual using HUME, a custom MATLAB toolbox. After staging, all epochs with artifacts and arousals were identified rejected by visual inspection before spectral analyses. Minutes in each sleep stage and sleep latencies (the number of minutes from lights out until the initial epoch of sleep, Stage 2, SWS, and REM) were calculated. Additionally, WASO was calculated as total minutes awake after the initial epoch of sleep, and sleep efficiency was computed as total time spent asleep after lights out (∼11:00 PM) divided by the total time spent in bed (∼11:00 PM to 9:00 AM) × 100. Sleep architectures were reported in *SI Appendix*, Tables S2 and S3.

### Heart Rate Variability.

ECG data were acquired at a 1,000-Hz sampling rate using a modified Lead II Einthoven configuration. We analyzed HRV of the R-waves series across the whole sleep/wake period using Kubios HRV Analysis Software 2.2 (Biosignal Analysis and Medical Imaging Group, University of Kuopio) according to the Task Force guidelines (53). R peaks (R waves of QRS signal on the ECG) were automatically detected by the Kubios software and visually examined by trained technicians. Incorrectly detected R-peaks were manually edited. Missing beats were corrected via cubic spline interpolation. Interbeat intervals were computed, and a third-order polynomial filter was applied on the time series in order to remove trend components. Artifacts were removed using the automatic medium filter provided by the Kubios software.

The HRV analysis of the RR series was performed by using a MATLAB-based algorithm. An autoregressive model (model order set at 16) was employed to calculate the absolute spectral power (ms^2^) in the LF HRV (0.04 to 0.15 Hz; ms^2^) and the HF HRV (0.15 to 0.40 Hz; ms^2^; an index of vagal tone) frequency bands as well as total power (TP; ms^2^; reflecting total HRV) and HF peak frequency (HFpf; Hz; reflecting respiratory rate). From these variables, we derived the HF normalized units (HF_nu_ = HF[ms^2^]/HF[ms^2^]+LF[ms^2^]) and the LF/HF ratio (LF[ms^2^]/HF[ms^2^]), an index often considered to reflect the sympathovagal balance (i.e., the balance between the two branches of the autonomic nervous system) but whose meaning has been recently put into question. The LF, HF, and TP measures had skewed distributions and, as such, were transformed by taking the natural logarithm. Since the LF-normalized units are mathematically reciprocal to HF_nu_ (i.e., LF_nu_ = 1 − HF_nu_), to avoid redundancy, only the HF_nu_ index is computed, an index often thought to reflect vagal modulation. Due to controversies about the physiological mechanisms that contribute to changes in LF activity, LF, LF/HF ratio, and HF_nu_ are difficult to make for these parameters, but they are reported for descriptive purposes.

In addition to the frequency domain parameters, RMSSD (ms;) was calculated as a measure of vagally mediated HRV in the time domain. Similar to the frequency adjustments, to adjust for skewed distributions in the RMSSD, we report the natural logarithm. Additionally, RR (ms; time interval between consecutive R-peaks, reflecting frequency of myocardial contraction) was calculated as an index of cardiac autonomic control in our analyses.

For time domain and frequency domain HRV measures during different sleep stages, consecutive artifact-free 5-min windows of undisturbed sleep were selected across the whole night using the following rules: a) the 1.5-min preceding and b) the entire 5-min epoch selected must be free from stage transitions, arousal, or movements. The windows were identified and averaged within Stage 2 sleep, SWS, and REM sleep. We also analyzed 5 min of presleep wakefulness (Rest). Epochs of N1 were not analyzed. All the HRV parameters by drug condition and sleep stage were reported in *SI Appendix*, Tables S4–S6.

### Power Spectral Analysis.

The EEG power spectrum was computed using the Fast Fourier Transformation. SWA (0.5 to 2 Hz), delta (1 to 4 Hz), theta (4 to 8 Hz), alpha (8 to 13 Hz), sigma (12 to 15 Hz), beta (15 to 30 Hz), and total power (0.3 to 35 Hz) were calculated for each sleep stage (Stage 2, SWS, and REM). The EEG epochs that were contaminated by muscle and/or other artifacts were rejected using a simple out-of-bounds test (with a ±200 µV threshold) on a high-pass–filtered (0.5 Hz) version of the EEG signals. Then, the normalized power spectra (% power of each frequency band of interest/total power) were averaged bilaterally within each sleep condition/stage/subject. Power analyses that showed significant drug effect were reported in *SI Appendix*, Tables S7 and S8.

### Effective Connectivity.

To explore the causal information flow between the central nervous system and the autonomic nervous system sleep features, we considered sigma to reflect CNS activity and HFln to reflect ANS activity. Sigma power of eight EEG channels (F3, F4, C3, C4, P3, P4, O1, and O2) and HF HRV were considered as signals to estimate effective connectivity. To adopt uniform timing across signals and avoid temporal misalignments between EEG signals and HF time series, a sliding window technique was incorporated with a window length of 5 min and stride of 5 s. All data during nighttime sleep was used to have continuous time series of sigma powers and HF, and a length of 5 min was selected to be consist with HRV process. Therefore, for each subject, nine different signals were constructed, including the ratio of sigma power band to total power of EEG of eight channels and HF power of HRV for each 5-min window (Fig. 3*A*).

Generalized partial direct coherence (GPDC) measure was used to estimate causal information flow between sigma power and HF. GPDC uses the multivariate vector autoregressive (MVAR) model to model causal interactions between signals and estimate directed causal information flow between signals by using the coefficients and parameters of MVAR.

After constructing sigma power and HF signals, GPDC was computed for each window with length of 500 samples (500 × 5 s = 2,500 s) with a stride of 250 samples. First, signals interactions were modeled by the MVAR model (Eq. 1):

[1] $$X\left(n\right)=\overset{p}{\underset{k=1}{\sum }}A_{k}X\left(n-k\right)+w\left(n\right),$$

in which X(n) is the vector of signal values (with a length of N, the number of signals, N=8) in time n, $X\left(n\right)={\left[x_{1}\left(n\right),\;x_{2}\left(n\right),\ldots ,x_{N}(n)\right]}^{T}$. p is the order of the MVAR model which was selected according to Akaike criterion, p=4. $A_{k}$ is the matrix of MVAR coefficients, and each element, $a_{ij}(k)$, stands how much j-th signal in time n−k affects i-th signal in time n, and w(n) is the vector of model’s additive Gaussian noise with zero mean and covariance matrix Σ. After modeling the interaction of the signals, GPDC was computed using frequency domain of coefficients and covariance matrix as the following:

[2] $${\bar{\pi }}_{ij}\left(f\right)=\frac{\frac{1}{{\text{Σ}}_{ii}}A_{ij}(f)}{\sqrt{\sum_{k=1}^{N}\frac{1}{{\text{Σ}}_{kk}{}^{2}}{|A_{kj}(f)|}^{2}}}.$$

Consequently,

[3] $$0\leq {|{\bar{\pi }}_{ij}\left(f\right)|}^{2}\leq 1$$

and

[4] $$\overset{N}{\underset{i=1}{\sum }}{|{\bar{\pi }}_{ij}\left(f\right)|}^{2}=1,$$

in which ${\bar{\pi }}_{ij}\left(f\right)$ is the estimated matrix of causal information flow and the j-th column represent causal information outflow from the j-th signal to all the other signals. Average values over frequencies were considered for further process, and based on the main purpose of the study, two quantifiers were defined as follows (Fig. 3*A*):

1. Causal information outflow from HF to all EEG channels, HF_Outflow_ – Average (*n* = 8) of causal information flow from HF to EEG sigma activity. HF_Outflow_ represents the strength of causal effect of HF to sigma power.
2. Causal information inflow to HF from all EEG channels, HF_Inflow_ – Average (*n* = 8) of causal information flow from EEG sigma activity to HF. HF_inflow_ represents the strength of causal effect of sigma to HF.
3. Effective connectivity ratio, HFInflow over HFOutflow, in which greater numbers represented a greater central sigma control over autonomic vagal activity than vice versa.

### Sigma/SO Coupling.

SO troughs were detected for each channel automatically using the algorithm introduced by Dang-Vu et al. (54). For each SO, the sigma power spectrum (12to 16 Hz) was computed in the time margin of SO trough to 1 s post-SO trough. To access SOs which were coupled with sigma waves, the median of all normalized sigma power of SOs for all recording was computed for each channel. The SOs which had sigma power greater than the median values in each quartile was considered as the SO–Sigma coupled, and the number of coupled SOs was considered to further statistical analysis.

### Statistical Analyses.

All statistical analyses were performed in R 3.6.2 using the libraries lme4 and lsmeans. *P* values less than 0.05 were considered significant, *P* values between 0.05 and 0.07 were considered trend significant, and *P* values greater than 0.07 were considered nonsignificant. We used a linear mixed model (LMM) to evaluate the effects of zolpidem on sleep architecture, EEG power spectrum, autonomic profiles, and behavioral improvements. LMMs were chosen because they allow modeling of random effects and allow for the intercept and slope to be correlated (55). LMMs are parametric models that use maximum likelihood estimates to obtain coefficients and covariance structures. LMMs do not depend on limited assumptions about variance–covariance matrix assumptions (sphericity). Additionally, LMMs allow inclusion of an unbalanced number of observations per participants in the analyses. Moreover, LMMs take into account the influence of factors whose levels are extracted randomly from a population (i.e., participants), thus yielding more generalizable results.

#### Sleep architecture and power spectrum.

Using LMMs, we tested for the main effect of drug condition for sleep architecture (*SI Appendix*, Tables S2 and S3), EEG power spectrum (*SI Appendix*, Tables S7 and S8).

#### Autonomic profiles.

For autonomic profiles, we tested for the main effect of drug condition and interactions between sleep stage and drug condition by approximating likelihood ratio tests (LRT) to compare LMMs with and without the effect of interest (56). We first built a reduced (nested) model with sleep stage as the only effect and then included drug condition as a fixed effect in the full model. By comparing the reduced and full model using the LRT, we can interpret if the drug condition significantly modulated the outcomes. Tukey’s correction for multiple testing was used for post hoc comparisons.

#### Effective connectivity.

Using LMMs, we tested for the main effect of drug condition, the main effect of inflow versus outflow, and the interaction between the two factors (Fig. 3 *B* and *C*). We first built a reduced (nested) model with inflow versus outflow as the only effect and then included drug condition as a fixed effect in the full model. By comparing the reduced and full model using the LRT, we can interpret if drug condition significantly modulated the outcomes. Tukey’s correction for multiple testing was used for post hoc comparisons.

#### Behavioral tasks.

To investigate the drug effect on cognitive enhancement, LMMs were used with the drug condition as the predictor of interest (fixed effect), the improvement in WPA and OS tasks as outcome variables, and participants as crossed random effects. As we assume larger individual differences of improvement and difference in improvement between drug conditions, our LMMs include both a random intercept and a random slope term. To account for practicing effect on the tasks, we included visit and baseline performance as a covariate in the models. We first confirmed no differences at baseline (Test 1) between the placebo and zolpidem visits (*SI Appendix*, Fig. S1). Next, we confirmed no differences of improvements across 12 h of waking (Test 2 to Test 1) between the placebo and zolpidem visits (*SI Appendix*, Fig. S1). We then tested the sleep-dependent changes in improvement: the overnight (Test 3 to Test 2) and 24-h (Test 3 – Test 1) changes (Fig. 4 *A* and *B*). Again, we tested for the effect of drug condition by approximating LRTs.

#### Correlations.

Lastly, we used a Pearson’s correlation coefficient to examine the functional roles of sigma, vagal activity, and causal information flow on sleep-dependent behavioral changes. We further used the Fisher r-to-z transformation to compare the differences between two correlations of interests.

## Supplementary Material

Supplementary File

## Data Availability

Data have been deposited at GitHub (https://github.com/pinchunc/sleep_switch_paper).
